# RETRACTED: Huang et al. Adenine Combined with Cisplatin Promotes Anticancer Activity Against Hepatocellular Cancer Cells Through AMPK-Mediated p53/p21 and p38 MAPK Cascades. *Pharmaceuticals* 2022, *15*, 795

**DOI:** 10.3390/ph18020263

**Published:** 2025-02-17

**Authors:** Jhen-Yu Huang, You-Cian Lin, Han-Min Chen, Jiun-Tsai Lin, Shao-Hsuan Kao

**Affiliations:** 1 Institute of Medicine, College of Medicine, Chung Shan Medical University, Taichung City 402306, Taiwan; 2 Cardiovascular Division, Surgical Department, China Medical University Hospital, Taichung City 404332, Taiwan; 3 School of Medicine, College of Medicine, China Medical University, Taichung City 404332, Taiwan; 4 Institute of Applied Science and Engineering, Catholic Fu Jen University, New Taipei 242048, Taiwan; 5 Energenesis Biomedical Co., Ltd., Taipei 114694, Taiwan; 6 Department of Medical Research, Chung Shan Medical University Hospital, Taichung City 402306, Taiwan

The *Pharmaceuticals* Editorial Office retracts the article “Adenine Combined with Cisplatin Promotes Anticancer Activity against Hepatocellular Cancer Cells through AMPK-Mediated p53/p21 and p38 MAPK Cascades [1]” cited above.

Following publication, concerns were brought to the attention of the Publisher regarding the overlap of an image with another publication representing different experimental conditions.

Adhering to our complaint procedure, an investigation was conducted by the Editorial Office and Editorial Board that confirmed that, a section of the Western immunoblot presented in Figure 3 (beta-Actin) overlaps, apart from a minor modification, with a section presented in a figure (Figure 4) in an earlier publication [2] from a different authorship group. While the authors cooperated with the Editorial Office during the investigation, they were unable to satisfactorily explain the presence of this overlap. As a result, the Editorial Board and Editor-in-Chief were unable to confirm the reliability of the findings and subsequently decided to retract this paper as per MDPI’s retraction policy (https://www.mdpi.com/ethics#_bookmark30, accessed on 15 April 2024) and in line with the Committee on Publication Ethics retraction guidelines (https://publicationethics.org/retraction-guidelines, accessed on 15 April 2024).

This retraction was approved by the Editor-in-Chief of the journal *Pharmaceuticals*.

The authors disagree with this retraction.
